# The bacteriome and mycobiome associated with oral squamous cell carcinoma: metagenomic analysis of samples from Yemeni and Sri Lankan cohorts

**DOI:** 10.1080/20002297.2017.1325188

**Published:** 2017-06-09

**Authors:** Nezar Al-hebshi, Manosha Perera, Irosha Perera, Deepak Ipe, Glen Ulett, David Speicher, Akram Nasher, Mohamed Maryoud, Husham Homeida, Ali Mohamed Idris, Tsute Chen, Newell Johnson

**Affiliations:** ^a^ Maurice H. Kornberg School of Dentistry, Temple University, Philadelphia, PA, USA; ^b^ School of Dentistry and Oral Health, Griffith University, Queensland, Australia; ^c^ Preventive Oral Health Unit, The National Dental Hospital (Teaching), Colombo, Sri Lanka; ^d^ Menzies Health Institute Queensland, Griffith University, Queensland, Australia; ^e^ Department of Oral and Maxillofacial Surgery, Faculty of Dentistry, Sana’a University, Yemen; ^f^ Department of Maxillofacial Surgery and Diagnostic Sciences, College of Dentistry, Jazan University, Jazan, Kingdom of Saudi Arabia; ^g^ Department of Microbiology, Forsyth Institute, Cambridge, MA, USA

## Abstract

There is increasing interest in the possible role of the oral microbiome in oral squamous cell carcinoma (OSSC). However, studies on the possible association between the oral bacteriome and OSCC remain inconclusive, while the potential role of the “mycobiome” in oral carcinogenesis has never been explored. I hereby present results from our recent studies on the bacteriome and mycobiome associated with OSCC based on analysis of samples from Yemeni and Sri Lankan cohorts. Tissue biopsies were obtained from the cases while deep buccal swabs or fibro-epithelial polyps were collected from the controls. Sequencing of the bacterial V1-V3 region of the 16S rRNA gene and the fungal ITS2 region using Illumina’s 2x300 bp chemistry was employed to study the bacteriome and mycobiome, respectively. Merged reads were classified to species level using a BLASTN-algorithm. Downstream analyses were performed using QIIME, PICRUSt, and LEfSe. *Fusobacterium nucleatum subsp. polymorphum, Pseudomonas aeruginosa, Prevotella* and *Campylobacter spp*. were more abundant in OSCC samples while *Streptococcus mitis* and *Rothia spp*. were overrepresented in the controls. Functionally, inflammatory bacterial attributes including bacterial mobility, flagellar assembly, bacterial chemotaxis and LPS synthesis were enriched in the tumors. Mycobiome analysis revealed a dysbiotic fungal community dominated by *C. albicans* in association with OSCC.

